# 卡瑞利珠单抗联合AVD方案一线治疗经典型霍奇金淋巴瘤的有效性和安全性

**DOI:** 10.3760/cma.j.issn.0253-2727.2022.05.015

**Published:** 2022-05

**Authors:** 可 赵, 华庆 王, 琳 李, 腾 宋, 书苹 李, 鸣寒 邱

**Affiliations:** 天津市人民医院肿瘤诊疗中心、天津市中西医结合肿瘤研究所、南开大学人民医院转化医学研究院，天津 300121 Department of Oncology, Tianjin Union Medical Center, Tianjin Cancer Institute of Integrated Traditional Chinese and Western Medicine, the Institute of Translational Medicine, Tianjin Union Medical Center of Nankai University, Tianjin 300121, China

ABVD方案（多柔比星+博来霉素+长春新碱+达卡巴嗪）是霍奇金淋巴瘤（HL）的标准一线治疗，但仍有不到30％的HL患者复发或出现耐药[1]–[2]，并且10％～20％的患者发生博来霉素相关肺毒性[3]–[4]。免疫检查点抑制剂的出现为淋巴瘤的治疗开拓了新视野，多项临床研究报道了程序性死亡受体1（PD-1）抑制剂在复发难治经典型霍奇金淋巴瘤（cHL）的出色疗效[5]–[7]。但迄今为止，免疫联合化疗方案用于HL一线治疗在国际上鲜有报道，国内更是缺乏研究。本研究我们回顾性分析2020年1月至2020年9月天津市人民医院收治的6例初诊cHL患者的病例资料，探讨卡瑞利珠单抗联合AVD方案（表柔比星+长春地辛+达卡巴嗪）（C-AVD方案）一线治疗cHL的有效性和安全性。

## 病例与方法

1. 病例：回顾性收集2020年1月至2020年9月就诊于天津市人民医院的6例初诊cHL患者的病例资料，所有患者均经病理及免疫组织化学确诊且临床资料完整。临床分期采用Ann Arbor分期，Ⅱ期（早期）、Ⅳ期（晚期）各3例。Ⅱ期HL参照2020年第2版NCCN指南[8]，具有红细胞沉降率≥50 mm/h、有B症状（发热、盗汗、体重减轻）、肿块最大径/胸腔最大径（MMR）>0.33、受累淋巴结区数>3个、包块直径>5 cm任意一项定义为具有不良预后因素；Ⅳ期HL根据国际预后评分（IPS）评估危险度。

2. 治疗方案：卡瑞利珠单抗200 mg/d，静脉注射，第0、14天；表柔比星35 mg·m^−2^·d^−1^，静脉注射，第1、15天；长春地辛3 mg·m^−2^·d^−1^，静脉注射，第1、15天；达卡巴嗪375 mg·m^−2^·d^−1^，静脉注射，第1、15天；4周为1个周期。3例Ⅱ期HL患者均接受4个周期的C-AVD方案，其中1例因初始表现为纵隔大包块化疗结束后接受放疗；2例Ⅳ期HL患者接受6个周期的C-AVD方案，1例Ⅳ期HL患者仅接受了4个周期的C-AVD方案。C-AVD方案结束后获得完全缓解的晚期患者接受单药卡瑞利珠单抗200 mg每月1次维持治疗，而治疗期间疾病进展者根据情况行二线治疗。

3. 图像分析：采用MEDEX软件对PET-CT原始图像进行分析，利用40％SUVmax为界值勾画感兴趣区体积，获得病灶的最大标准化摄取值（SUVmax）、肿瘤代谢体积（MTV）和总糖酵解量（TLG），在CT图像上测量最多6个靶病灶的垂直径乘积之和（SPD）。

4. 疗效及不良反应评价：疗效评价标准采用Lugano修订的2014版标准[9]，分为影像学缓解（CT/MRI评效）和代谢缓解（PET-CT评效）。不良反应根据美国国立癌症研究所常见不良反应评价标准5.0版进行评价。

5. 随访：采用电话、门诊或者住院病历查阅的方式进行随访，末次随访时间为2021年6月15日，中位随访时间12.3（9.5～17.5）个月。无进展生存时间（PFS）定义为自确诊后首次用药至首次肿瘤发生进展或复发、患者因任何原因死亡的时间。

## 结果

1. 一般临床特征：6例初诊cHL中，结节硬化型4例（66.7％），混合细胞型2例（33.3％），男女各3例，中位发病年龄为47（34～64）岁。以浅表淋巴结肿大起病者最为常见，其他首发症状包括纵隔包块、发热。2例有B症状，Ⅱ期具有不良预后因素者3例。EBER阳性者1例（16.7％），EBER阴性者5例（83.3％），无一例外周血EBV-DNA阳性。具体临床资料见表1。

**表1:** C-AVD方案治疗6例初诊经典型霍奇金淋巴瘤患者的临床资料

例号	性别	年龄（岁）	病理类型	原发部位	分期	B症状	预后不良因素	IPS	EBER	EBV-DNA	一线治疗	维持治疗
1	男	34	混合细胞型	淋巴结	Ⅱ	无	ESR≥50	-	阳性	阴性	C-AVD×4	无
2	女	64	结节硬化型	淋巴结	Ⅱ	有	B症状	-	阴性	阴性	C-AVD×4	无
3	女	57	混合细胞型	纵隔、淋巴结	Ⅱ	无	MMR>0.33、ESR≥50	-	阴性	阴性	C-AVD×4	无
4	女	34	结节硬化型	淋巴结、右肺、肝、脾、骨	Ⅳ	无	-	3	阴性	阴性	C-AVD×6	C×4
5	男	38	结节硬化型	淋巴结、骨	Ⅳ	无	-	3	阴性	阴性	C-AVD×6	C×10
6	男	64	结节硬化型	肝脏、淋巴结	Ⅳ	有	-	6	阴性	阴性	C-AVD×4	无

注：B症状：发热、盗汗、体重减轻；ESR：红细胞沉降率；MMR：肿块最大径/胸腔最大径；IPS：国际预后评分；EBV：EB病毒；C-AVD方案：卡瑞利珠单抗（C）+表柔比星（A）+长春地辛（V）+达卡巴嗪（D）；-：不适用

2. 疗效评估：全部6例患者中，C-AVD方案治疗结束后5例获得完全缓解（CR），1例（例5）应用C-AVD方案2个周期后获得CR，但在治疗4个周期后发生疾病进展。客观反应率（ORR）和CR率均为83.3％。所有获得CR的患者截止到末次随访仍维持CR状态。全部患者基线MTV及TLG平均水平分别为53.49 cm^3^和290.03 g。一线治疗结束时，所有获得CR的患者SPD减轻超过50％，MTV和TLG较基线下降超过80％（表2）。

**表2:** C-AVD方案治疗6例初诊经典型霍奇金淋巴瘤患者的疗效与不良反应

例号	疗效	MTV（cm^3^）		TLG（g）		SPD（cm^2^）		不良反应
		基线	治疗后	基线	治疗后	基线	治疗后	
1	CR	41.80	3.97	116.50	9.09	21.50	2.29	白细胞减少、中性粒细胞减少、疲乏、恶心
2	CR	7.92	0.00	57.00	0.00	9.00	0.60	白细胞减少、中性粒细胞减少、贫血、疲乏、恶心、呕吐、心动过速、高血压
3	CR	59.29	6.69	448.88	12.95	35.86	0.50	白细胞减少、中性粒细胞减少、贫血、疲乏、恶心
4	CR	93.58	15.04	493.10	17.20	39.55	2.10	白细胞减少、中性粒细胞减少、贫血、疲乏、输注相关反应、恶心、呕吐、甲减、齿周炎、转氨酶升高
5	CR	87.31	14.27	487.50	24.60	31.72	10.94	白细胞减少、中性粒细胞减少、贫血、疲乏、输注相关反应
6	PD	31.04	-	137.20	-	19.05	2.88	白细胞减少、中性粒细胞减少、贫血、疲乏

注：CR：完全缓解；PD：疾病进展；MTV：肿瘤代谢体积；TLG：总糖酵解量；SPD：多个靶病灶垂直径乘积之和；C-AVD方案：卡瑞利珠单抗（C）+表柔比星（A）+长春地辛（V）+达卡巴嗪（D）；-：不适用

3. 不良反应：常见的不良反应见表2，以血液学毒性较为常见，常见非血液学相关不良事件为疲乏和恶心。除2例患者发生3～4级白细胞减少外，其他不良反应均1～2级。经过对症处理后可缓解且不影响后续治疗。与卡瑞利珠单抗相关的主要不良事件为输注反应和甲状腺功能减退，所有患者均未发生反应性皮肤毛细血管增生症（RCCEP）及免疫相关性肺炎等其他免疫相关不良事件。

## 讨论

作为我国自主研发的PD-1抑制剂，卡瑞利珠单抗治疗复发难治cHL 的ORR为76％，CR率达28％[7]。鉴于其在复发难治cHL展现出积极的有效性和安全性，已在国内获批用于复发难治cHL的三线治疗。就HL的一线治疗而言，化疗联合放疗的地位仍不可撼动，其5年OS率约90％。但由于放化疗所带来的心肺损伤和第二肿瘤的发生，平衡治疗有效率和远期毒性就显得至关重要[10]–[11]。有研究者探索将免疫治疗用于HL的一线治疗，希望能够增效减毒。然而，有关PD-1抑制剂联合化疗用于cHL一线的治疗仅有少数报道，国内尚无相关研究。

本研究回顾性分析了我科室6例初治cHL一线治疗使用C-AVD方案的数据资料。结果显示，卡瑞利珠单抗联合AVD方案在cHL一线治疗显示出较高的ORR和CR率。与传统的ABVD方案及ECHELON-1研究报告的靶向化疗方案相比[12]，去除博来霉素的C-AVD方案似乎并不会对cHL的治疗效果产生负面影响。NIVAHL研究[13]报道Nivolumab联合AVD方案同步或序贯治疗预后不良的早期cHL的疗效，联合组和序贯组ORR分别为100％和98％，CR率分别为81％和86％。CheckMate205研究报道了Nivolumab联合AVD方案在晚期cHL患者CR率达到67％[14]。本研究早期与晚期病例的缓解率与上述研究一致，提示卡瑞利珠单抗疗效可能并不亚于Nivolumab，疗效较为可观。对比免疫单药在复发难治cHL后线治疗中的疗效，与Nivolumab和Pembrolizumab相比，卡瑞利珠单抗在复发难治cHL三线治疗的有效率更高[5]–[7]，且该研究的目标人群均为中国患者，因此卡瑞利珠单抗可能是更适合中国人群的免疫检查点抑制剂。

本研究中，晚期cHL患者中有1例患者C-AVD方案一线治疗4个周期后发生了疾病进展。该患者是老年、高危晚期cHL患者，初次就诊于我科时B症状明显，伴有严重贫血及低蛋白血症，且由于肿瘤累及肝脏肝功能严重受损，IPS评分高达6分。患者一线应用C-AVD方案2周期后很快获得了CR，且各项指标基本恢复正常。后续治疗继续应用该方案，除新发骨转移外，余病灶仍维持缓解状态。该例患者发生疾病进展可能与肿瘤异质性有关，同时也提示老年高危cHL患者预后较差，需要根据病情及时调整治疗方案。

^18^F-FDG PET-CT目前被常规应用于HL的分期及疗效判定，对预后的判定也具有一定的应用价值[15]–[16]。研究表明，MTV及TLG是衡量生物活性肿瘤负荷的潜在指标，能够对预后不良的HL进一步进行风险分层[17]，且基线MTV和TLG可预测HL患者的治疗反应和生存时间[18]。我们的研究评估了MTV和TLG作为HL一线免疫化疗后治疗反应评估的生物标志物，结果显示一线C-AVD方案治疗后获得CR的5例患者，治疗后的MTV和TLG 接近完全减少，与基线相比，MTV和TLG下降超过80％，与Pembrolizumab序贯AVD方案结果接近[19]，该部分患者随访至今未发生疾病进展。而另1例发生疾病进展的晚期cHL患者，基线MTV和TLG水平较高，预示着较高的疾病进展风险，与文献[18]研究结果一致。MTV和TLG可能是量化治疗反应的有效参数。

从安全性来看，C-AVD方案最常见的不良反应为1～2级的血液学毒性、疲乏和恶心，这与单药卡瑞利珠单抗[7]和AVD方案[20]相关历史研究保持较高的一致性。此外，本研究全部患者均未发生免疫相关性肺炎亦或是化疗相关肺毒性，C-AVD方案似乎具有较好的肺部安全性。与同类PD-1抑制剂相比，应用卡瑞利珠单抗RCCEP的发生率较高，但联合化疗或抗血管生成药物后，RCCEP发生率显著降低[7],[21]。本组病例未发生RCCEP，这可能与联合AVD化疗方案有关。本研究所报道的不良反应亦与NIVAHL研究[13]、CheckMate205研究[14]报道相似，没有出现新的既往未曾报道过的不良事件，表明该免疫化疗方案的安全性良好。

综上所述，本研究结果显示卡瑞利珠单抗联合AVD方案一线治疗cHL疗效可观，安全性良好。本研究为小样本回顾性研究且随访时间较短该方案是否可以广泛应用于临床仍需要进一步探索。
